# Academy of Stomatology

**Published:** 1898-10

**Authors:** 

**Affiliations:** Academy of Stomatology


### Reports of Society Meetings.

### ACADEMY OF STOMATOLOGY.

A REGULAR meeting of the Academy of Stomatology was held April 26, 1898, at the rooms of the society, 1731 Chestnut Street, Philadelphia, the President, Dr. Edwin T. Darby, in the chair.

A paper entitled "Treatment of Cervical Borders" was read by Dr. Safford G. Perry, of New York.

(For Dr. Perry's paper, see page 567.)

Upon the conclusion of the reading Dr. Perry showed the illuminating devices alluded to in his text, and also demonstrated the use of the pluggers, which he regretted that he had forgotten to bring with him.

### DISCUSSION.

*Dr. Guilford.*—I am very much gratified, indeed, to have Dr. Perry come over and read us his able paper. He rather apologizes towards the last for going too much into detail. I think the mistake is generally made in the opposite direction. I am very glad, indeed, that he said all he did say.

We formerly heard a good deal about the failure of gold fillings at cervical borders, and it was inferred by some that they failed simply because they were of gold. The fact of the matter is that a large share of these failures were due to the fact that the cavities were not properly prepared. That they were not was partly because we did not understand how to prepare them, and partly because we did not use the rubber dam, and did not previously dry the cavities thoroughly. In the preparation of a cavity without the rubber dam these little checks at the cervical margin are not noticeable, but when the cavity is thoroughly dried we can see them. These places should always be cut out. I meet with them every day in my practice, and I keep cutting until I remove every portion. I think we have fillings that are more satisfactory than those of twenty-five years ago, simply because we know now better how to prepare cervical margins. It was not very many years ago that Marshall Webb and others thought that, in order to get a cavity properly prepared at the cervical margin, it was necessary to go above the free margin of the gum, and to extend the cavity upon the sides, in order that it might be cleansed by the brush and cheek. In that way we made cavities that were inordinately large. I have felt that this was not necessary, and I am glad that Dr. Perry does not believe in extending cavities beneath the gum for the sake of getting them beyond the danger line. If I find that the edge crumbles I cut down farther, but when I get a sharp margin I stop, no matter whether it is under the gum or not. I have noticed that within the last ten or fifteen years, not only with my own but with the fillings of others, that there are far fewer failures at cervical margins of gold fillings than there formerly were. I think this is due to the proper preparation of the cavity, better quality of gold, or perhaps better manipulation of the gold, and then, too, the care exercised in the finishing of the fillings.

In the placing of fillings with the use of the rubber dam, clamps, matrices, etc., we must necessarily cause a certain amount of pain, but we should not exceed the minimum amount. The pain of finishing the filling is minimized by the use of the matrix. It should be flexible and sufficiently yielding to permit the filling to be built out to the same shape as the tooth, but a little beyond the cavity margin. All that is then needed is to trim it up with a suitable sharp instrument, such as the Gardiner knives, and to polish it. This method of finishing is almost painless, while the old method of filing the filling to shape caused much suffering. Finally, the ordinary paper disk or tape properly used will make the surfaces very smooth indeed.

*Dr. McQuillen.*—I want to thank Dr. Perry for his very valuable paper, and also for the many instruments that he has given us at different times. I am always particularly pleased to hear him. The method of repairing gold fillings with amalgam is a practice I have carried out for a great many years, and I treat the work of other men as I treat my own; it never seemed to me an honest thing to tear out a filling that had failed at the cervical margin when it could be patched and saved for years. I have had very good results from these repairs.

*Dr. Kirk.*—I think every one has been very much interested in this paper. There were two points that occurred to me during its reading that seemed to be especially interesting: for one thing, the emphasis that Dr. Perry has laid on the well-known habit of zinc phosphate cements to disintegrate at the cervical border. Of course, as every one knows, zinc phosphates are a heterogeneous lot: they have all sorts of variations of physical properties and are more or less valuable; but even supposing zinc phosphate to be a constant thing without variations in itself, the conditions under which it is placed in the mouth vary so considerably that in some mouths it may do well and in others it may absolutely fail.

Attention has been brought recently by Dr. Cassidy to a fact that has not been recognized in relation to oxyphosphate of zinc, and that is its liability to disintegrate under the action of alkalies.

More recently a gentleman, whose name I cannot recall, wrote a little note for publication in reference to ammonia as a means of disintegrating zinc phosphate cements from about the broken pins of artificial crowns. He recommends the application of ammonia water to the zinc phosphate cement, and says it rapidly brings about its disintegration. I have made experiments myself, and find that zinc phosphate can be disintegrated by the application of ammonia. It seems that the phosphoric acid leaves its combination with the zinc, uniting with the ammonia, and the compound falls apart.

Dr. Cassidy explains the disintegration of zinc phosphate at the cervical margins upon the hypothesis that putrefaction of nitrogeneous matter takes place there, and that ammonia is produced. Whether that be true I do not know. It is evident that where the alkaline condition is pronounced it would be possible for zinc phosphate fillings to disintegrate. I have seen cases where the failure of cervical margins with zinc phosphate has not been a failure of the cement, but of the tooth substance itself. I have seen caries supervene at the cervical margin, where the imprint of the original margin was still in the zinc phosphate. So not all failures of zinc phosphate are due to disintegration of zinc phosphate. I do not say that is frequent, but I have seen such fillings where the imprint was left.

There is another feature of Dr. Perry's paper that seems to me to need further emphasis, and that is the question of failures of fillings at the cervical margin by over-malleting. Many have fallen into the error of believing that cohesive gold requires an excessive amount of force to secure its consolidation. Two things have led me to a different view in regard to that matter: first, the demonstration which I saw given in New York by Dr. Shumway, of absolutely welding cohesive gold-foil by simply the wiping motion of ivory points. One piece of gold-foil was put upon another in this manner, and the mass afterwards tested and found to be solid and homogeneous. Second, the demonstration by Dr. Gardiner to-day, where he packed Nos. 60 and 90 rolled gold with as light a blow as I ever saw used on an electrical mallet. I felt as though the force was not sufficient to securely weld the piece, but when I saw him whittling down from the margins towards the filling, giving it as severe a test as possible,—and the gold was cut off in shavings, —I was convinced that he had produced an absolutely homogeneous result. The danger of fracturing the cervical margin was reduced to a minimum.

*Dr. Pierce.*—I do not know that I can add anything. I have been very much interested in this paper. I noticed that the essayist and others spoke of the enamel of the cervical border.

Nearly all our cervical borders reach to the cementum, and I think that is one reason why this locality is a complicated one, in which it is much more difficult to make a reliable operation.

I feel oftentimes that gold fillings, instead of being cut out, can be patched very satisfactorily by the use of amalgam, when we can make a little undercut above the filling.

Years ago Dr. Neall handed me a tooth he had extracted ; above the gold filling he had placed an amalgam repair, saving the tooth satisfactorily, although the tooth subsequently loosened and came out. I believe many operators have utilized amalgam in that way very satisfactorily.

I am convinced that our operations are made more valuable by the use of good antiseptics in the mouths of our patients, and that many operations that would formerly have failed by virtue of unfavorable conditions, and also the sound teeth, have been protected by the increased hygiene of the oral cavity. I scarcely believe that the credit is due to better operations.

I do not think that cohesive gold has added anything to the success of our operations upon the cervical border; I think it has detracted from it. The success has been due to the care subsequently given to the teeth by the patient.

*Dr. Register.*—I was pleased to hear Dr. Perry condemn deep undercuts and state that they sever the dentine fibrils and permit blue discoloration about fillings. I always shape my cavities so that such a condition will not exist.

I believe in the matrix, and use thin strips of soft metal, such as German silver or copper, which I ligate to the tooth with floss silk. By the use of a matrix a compound cavity is converted into a simple one to our manifest advantage. Inasmuch as many cavities may be prepared carefully and yet upon examination with a glass show certain defects of the margin, it is necessary to carefully examine for any such defects and to use antiseptics to destroy any germs which may be located upon the margins or walls. To this end I use seventy-five per cent. nitrate of silver, as suggested by Dr. Truman, but as that stains the tooth, I change the nitrate after it has acted upon the germs into iodide of silver by the action of iodine. In turn this may be destroyed by ammonia, leaving the tooth sterile and stainless.

*Dr. James Truman.*—There were one or two points that arrested my attention during the reading of the paper that seemed to me to be of importance.

I have a very great fear of zinc phosphate, not only at the cervical borders, but upon the floors of deep cavities, where we are using it oftentimes with danger to the pulp, as it must certainly affect the odontoblastic processes in the dentine. There is too much carelessness in its use. I have recommended the students in the clinics, whenever I find them using oxyphosphate of zinc at cervical borders or upon the floors of cavities, to lay a protection of gutta-percha beneath it.

When I heard Dr. Perry state that he could not use tin at the cervical border without its disintegrating, it seemed strange to me how we all differ on many points that are of practical importance. I remember that Dr. Jenkins, of Dresden, once said that he could not fill a cervical border with gold, that he had given that up as a practical impossibility, but that he could use tin and gold with perfect satisfaction, and I am forced to agree with him, not only as to the use of tin and gold, but tin alone, when put in upon the cohesive principle, as I think all tin should be used. Of course, I bow always to Dr. Perry's large experience in this matter, and hesitate even to oppose that much of his paper.

I believe a large proportion of failures at this time are due to the extravagant use of matrices and supporters, rubber dams, ligatures, and everything else that go to make up the filling of the present time. I think the dentist, as he leaves a filling, pays very little attention to subsequent results, and that a large proportion of cases of pyorrhœa have their origin in the operator himself. The gingival margins are left in a state of irritation which develops into inflammation and pyorrhœa. I had an illustration of that in the clinic at the University, where one of the students had placed the day before his rubber dam and ligatures without previously antisepticizing the parts (which I had always urged), and the result was a high degree of inflammation, not only around the tooth operated upon, but also around the next tooth. I should have been very glad if Dr. Perry had spoken more at length on that particular point. I regard the cervical border, and always have, as he does, as the delicate point of operating. I do not regard it, however, as impossible to place cohesive gold there. In an earlier period, after previous years of use of soft foil, we came to the exclusive use of cohesive gold. We saved cervical borders, I think, quite as well as before. I saw Dr. Gardiner operate to-day, and I must say that I was very much pleased with the blow of his mallet. Still I do not believe in using one at the cervical border, as I am satisfied that to its excessive use is due much of the trouble with that border.

Cracks can be made by over-malleting, and when made at the cervix invite decay.

In olden times when cohesive gold was first introduced, and we had no mallets but used simply hand-pressure, the cervical border remained intact. I have great faith in combination fillings, and I believe that by the use of amalgam and gold, or the various combinations of gutta-percha, zinc phosphate, amalgam, and gold, more teeth will be saved than formerly.

*Dr. Gardiner.*—I feel greatly indebted to Dr. Perry for his very conservative paper. I came expecting to get very many ideas and I have done so.

I do not think it is necessary to extend proximal cavities beneath the gum. I think they should be cut beyond the point of contact, but that does not indicate that they should be extended farther if the tooth is in good condition. I think cohesive gold can be packed against cervical walls perfectly, provided it be done in the proper way, either by hand-pressure or mallet. I use cohesive gold to-day simply because I get better results from it. That does not necessarily follow it would be better for everybody else. I use a great deal of leaf gold at the cervix, because I think it is extremely soft and can be packed thoroughly with very little trouble, either by hand-pressure or mallet. I use a mallet for convenience; there is less danger of slipping, and I think in my hands less danger of fracture of the wall than with hand-pressure.

In relation to oxyphosphate fillings, it has been my practice to put gutta-percha next to the gum. I have had excellent results from using high-heat gutta-percha, and prefer it to low-heat, because there is less liability to bulge.

*Dr. Louis Jack.*—In deference to the views of Dr. Abbot, of Berlin, and of others who have followed his opinions, I have repeatedly used tin at the cervical border of fillings. The result has been that the tin has wasted, leaving a depression in the tin at the cervix. This waste I attributed to electrolysis, since the tin obtained the relation of a positive element to the gold. Next I mixed the gold and tin by folding the two together, and applied it in cases where the teeth presented but slight resistance to the exciting causes of caries. The results here were better, since the same waste did not appear. Some peculiar action takes place by which the two metals appear to combine to form a dark mass, not having much hardness. I think, however, that at length this mass must become disintegrated. To my mind it has merely its softness to recommend it, and yet it is hardly superior to non-cohesive gold in this respect. I am decidedly of the opinion that there is no incompatibility between gold and tooth-structure if the method of filling is such as will produce perfect contact between them, and at the same time sufficient density to retain smoothness. I have had gold fillings continue in this position side by side with plastics where the latter failed for one cause or another, probably because the latter did not present the smooth surface essential to cleanliness. At the cervical border I prefer, first, absolutely non-cohesive gold, and, second, a good amalgam. The latter does not appear to undergo the changes which take place with tin.

*Dr. Roberts.*—I would like to ask Dr. Perry whether his plugger-points are flat or rounded on the ends. In the place in which he uses that triangular plugger-point I have been using the flat-edged-knife plugger-point.

I am also glad of the very good word that Dr. Perry gave for gutta-percha; I think success in packing gold lies in being kind to it. I think that is the chief feature of Dr. Gardiner's manipulation of heavy foil,—he is kind to his gold, and handles it gently.

*Dr. Perry.*—These pluggers are made perfectly flat upon the plugging end, which is triangular in form, and slightly serrated. The sharp corners do not count for so much; it is the peculiar rake of the point of the instrument, which enables it to go against the matrix, that is the telling thing.

*Dr. O. E. Inglis.*—It may possibly be superfluous to say anything more, but as the subject of combination fillings has been brought up, I would state that a year ago I saw a combination filling made of gold and amalgam which had been in place for seventeen years. It was in a position in which gold would have served equally well, but it was doing very good service.

*The President.*—Dr. Perry, have you anything to say?

*Dr. Perry.*—Just a word, Mr. President. I am obliged to Dr. Peirce for calling attention to the cervical margin above the junction of the enamel. I thought after the paper was finished that I had not given it attention. Dr. Guilford's remarks remind me of the subject of careful preparation of these cavities.

In reference to the wasting away of tin-foil fillings at the cervical margin, my own belief is that not in all mouths does tin-foil disintegrate, but that it usually does so at the cervical margins after a few years. In some mouths I am certain of it, and I believe myself that tin foil dissolves the same as oxyphosphate does. Of course, we know perfectly that when tin-foil is exposed to wear it always remains bright, and is one of the best fillings we have, but those soft tin-foil fillings slowly disintegrating at the cervical margin, while the rest of the filling stands well, I have seen for many years, and so continuously that I have come to be naturally distrustful of tin when used in these places, and for that reason I have of late years eschewed its use at the cervical margin, either alone or combined with gold; with the combination of tin and gold I obtained no better results than with tin. I could get as good a margin with non-cohesive gold (rolled) as I could with the combination.

*Dr. James Truman.*—If tin is packed by the old method, then it does disintegrate, but if used cohesive as it is now made, I think it will stand as well as most materials. It must be packed upon the cohesive principle and must be of good quality. As formerly made, with the oxide left on the surface and packed in ropes upon the wedge principle, the results Dr. Perry speaks of would be liable to follow.

*Dr. Perry.*—Mr. President, I may be wrong in this matter. I remember that when you came over and read a paper before our society you brought with you some teeth that had been filled with tin by your students, and I remember that years ago we saw in New York tin fillings made by you from fresh-cut shavings of block tin, and that these seemed to possess a solidity and hardness we had never seen before. It may be that I have been misled, and that the tin used then by myself, and fillings I had seen placed by others, may have been put in in the loose way and disintegrated for that reason.

The other day, in a clinic before the Odontological Society, Dr. Shumway demonstrated several new points. In the first place, he annealed tin over mica nearly to the point of its melting, and packed it into the bottom of the cavity in the usual way. That tin had the appearance of being a solid piece, and any one upon seeing the operation must have felt at once that the heat used had much to do with the solid condition obtained. He seemed to punch it into the cavity very easily without any effort, and as soon as an ivory point was rubbed over it there came out a beautiful surface; then a little flat piece of cohesive gold gently touched with the ivory point was run back and forth. The gold was brought into adhesion to the tin. If that style of filling teeth could come into vogue, I think we could feel that we were artists. It is very far removed from the blacksmith's sledge-hammer method of hammering gold.

I am glad also to commend Dr. Gardiner's idea of packing with very light blows of the mallet, and I believe that can be done. I have done it myself and in about that way. I have found a great many times that cohesive gold could be very accurately and easily packed by gently tapping it. If you pack it too much you will harden it. In fact, it is claimed that flexibility and softness of the ivory point, together with a peculiar elasticity of the handle of the instrument, are the very conditions necessary to impart that surface to the gold which allows the next piece that comes on to cohere, while if the gold is rubbed with hard steel instruments the surface is so hard that the next piece of gold coming into contact does not cohere.

Dr. Kirk saw some of Dr. Shumway's work, and, I think, felt it was a surprising thing to see what he did.

*Dr. Kirk.*—I am full of this matter. I do not want to interrupt, but I am sure that the matter which Dr. Shumway has developed is important. Whether it will develop as a practical method of operating is another question, for we have had various methods introduced that have been extremely interesting, but which have fallen into disuse from a practical stand-point; but it was interesting to me as a practical demonstration of the scientific exposition by Dr. Black, that where we have a clean surface of gold, as soon as we get contact we have cohesion. There is no doubt of it, and Dr. Shumway has demonstrated that it requires practically no force to bring the tin and gold surfaces into close relationship, that we get perfect adhesion of these materials. I believe his argument is based on correct principles.

Upon motion by Dr. Register a vote of thanks was presented to Dr. Perry for his paper.

Adjourned.

OTTO E. INGLIS,
*Editor Academy of Stomatology.*

